# Notes and News

**Published:** 1905-10-21

**Authors:** 


### Notes and News.

It is probable that the old naval hospitals of America will shortly be remodelled and renovated. An official scheme has been drawn up which includes considerable enlargement.

There is to be an International Exhibition in Milan in April 1906. The British Government has made a grant of £10,000 for the creation of a British section. There will be special sections for hygiene and social economy.

By the death of Earl Fortescue the North Devon Infirmary has lost a good friend. He has stimulated interest in the institution to a great extent and his son who succeeds to the title has already rendered personal service to it. He took part in the planning of the new sanitary annexes which are to be added to the infirmary.

The cottage hospital at Harrow is about to be enlarged. Of course it is often difficult to estimate the growth for the direction which population will take when a hospital is first built in a country district. Harrow has suffered through this difficulty. The hospital is badly situated and the site at its disposal is far too small to accommodate the enlargement and provide sufficient surrounding space.

We draw attention to the excellent suggestion made by Mr. Walford in our "Letter Box" this week. The covered way is always attractive and useful, and as a matter of convenience it has been recognised by the Brixton authorities, who have provided such excellent arcades for shoppers. The health of the people is equally worthy of consideration.

A new difficulty for the working man is threatened. In Paris lately several maladies have been traced to hair-dye; eye affections and headaches are amongst the complaints which are traced to the use of dye. Aniline products were found in one of the dyes which appeared to be harmful, and no doubt cheap hair-dye might contain aniline colouring, and such should certainly not be used.

An anonymous donor has presented £600 to St. Bartholomew's Hospital Medical School Research Fund.

At the last meeting of the Metropolitan Asylums Board it was reported that the number of fever cases since the beginning of August exceeded by 1,256 those of the previous year. The cases were reported to be increasing rapidly.

Mr. Henry Morris, F.R.C.S., the senior surgeon of the Middlesex Hospital, has made the munificent gift of £1,000 to form the nucleus of a permanent endowment fund for the maintenance of the medical school of the Middlesex Hospital.

Some time ago the Town Council of Brighton opened a pavilion of ten beds at the Borough Sanatorium for the use of consumptives. The result is reported as being so good that 40 per cent. of the patients have returned to their occupation after treatment. The medical officer for health recommends that thirty beds should be available. There is a local trust which is prepared to assist in the work.

It is suggested that Limerick should have a consumption sanatorium. At the meeting convened to discuss the question, a guardian remarked that the chief cause of consumption was overcrowding, and that steps should be taken to remedy this, and then there would not be much difficulty in grappling with the disease. Another guardian seemed to regard expenditure as of the first importance, and said, "The only thing we have to do is to guard against an increase in the rates."

On October 21, Trafalgar Day, a great centennial celebration is to take place in the Albert Hall, arranged by the British Soldiers' and Sailors' Society. There will be an organ recital, and afterwards there will be several interesting events, including a cinematograph exhibition of our Navy from 1805 to 1905. The "Death of Nelson" will be sung by Mr. Ben Davies, when the Union Jack will be hoisted half-mast high. In the evening a Nelson centenary concert will take place. Tickets can be procured from the Society at 680 Commercial Road, E., and also the little Nelson mementoes sold at 1s. which contain *Victory* copper.

During the past summer some members of the Yorkshire Automobile Club placed twenty-five motor-cars at the disposal of the patients at the Huddersfield Infirmary. Those patients who were able to take advantage of the opportunity, were driven to the Moors, where a good meal was provided for them. The matron, house-surgeon, secretary, and twenty nurses accompanied the party. The excursion proved a great success, and it is to be hoped that the idea will be adopted in other parts of the country. Patients at convalescent and incurable homes would benefit by such kindness, even more than the more limited number of convalescents at a general hospital.

